# Antioxidant Characterization and Biological Effects of Grape Pomace Extracts Supplementation in *Caenorhabditis elegans*

**DOI:** 10.3390/foods8020075

**Published:** 2019-02-15

**Authors:** Begoña Ayuda-Durán, Susana González-Manzano, Irene Gil-Sánchez, M. Victoria Moreno-Arribas, Begoña Bartolomé, Marisa Sanz-Buenhombre, Alberto Guadarrama, Celestino Santos-Buelga, Ana M. González-Paramás

**Affiliations:** 1 Grupo de Investigación en Polifenoles, Universidad de Salamanca, Campus Miguel de Unamuno, 37007 Salamanca, Spain; bego_ayuda@usal.es (B.A.-D.); susanagm@usal.es (S.G.-M.); paramas@usal.es (A.M.G.-P.); 2 Institute of Food Science Research (CIAL), CSIC-UAM, C/ Nicolás Cabrera 9, 28049 Madrid, Spain; irene.gil.sanchez@cial.uam-csic.es (I.G.-S.); victoria.moreno@csic.es (M.V.M.-A.); b.bartolome@csic.es (B.B.); 3 Bodega Matarromera, S.L. Ctra. San Bernardo s/n. Valbuena de Duero, 47359 Valladolid, Spain; msbuenhombre@gmail.com (M.S.-B.); imasd@emina.es (A.G.)

**Keywords:** grape pomace, *C. elegans*, oxidative stress, ROS, lifespan, antioxidant activity

## Abstract

The aim of this work was to evaluate the biological activity of four grape pomace (GP) extracts that are rich in polyphenols using *C. elegans* as an in vivo model. Different concentrations of the GP extracts were assessed for their effects on the resistance of *C. elegans* against thermally induced oxidative stress, accumulation of reactive oxygen species (ROS), and lifespan. The cultivation of *C. elegans* with relatively low concentrations of GP extracts increased their resistance against thermal stress and prolonged their lifespan, while high levels displayed detrimental effects. In the studied extracts, maximum protection was observed for levels of polyphenols around 7 to 9 µg gallic acid equivalents per cultivation plate. The obtained results suggested that small changes in the ROS levels could have beneficial effects, although further studies are required to fully understand the impact of the extracts and assayed doses on ROS levels to explain the mechanism that is involved in the observed effects.

## 1. Introduction

Phenolic compounds have been the center of attention of numerous studies, as they have been related with the putative beneficial effects attributed to moderate consumption of wine in the prevention of some chronic pathologies, such us cardiovascular diseases, neurodegenerative disorders, atherosclerosis, and cancer [1]. These compounds have their origin in the grape, and only part of them is transferred to the must during winemaking. As a result, important quantities of phenolic compounds still remain in the grape pomace (GP). This by-product consists of solid wastes of seeds, skins, and stems that suppose a serious economic and environmental problem due to the huge quantity that is produced. However, the grape pomace has been considered to be cheap source for the extraction of antioxidant flavonoids, which could be used in dietary supplements and for cosmetic or pharmaceutical purposes [2].

Aging is a degenerative process that is caused by a variety of molecular and cellular sources of damage. However, it is well documented that irreversible oxidative damage accumulates during aging [3]. The discovery of lifespan modulation phytomolecules may promote the development of natural therapies against age related afflictions [4]. In this sense, numerous lines of evidence suggest that dietary polyphenols have the capacity to mitigate age-associated cellular damage that is related to oxidative stress, chronic inflammation, and toxin accumulation. As there are multiple diseases and disorders that are related with aging, the need to achieve a whole understanding of the role that polyphenols might have in modulating them is critical [5].

*Caenorhabditis elegans* is a free-living soil nematode that presents small size, short life span, high reproduction rate, and well-defined genetic pathways [6,7]. Furthermore, it is estimated that 60–80% of the *C. elegans* genes have a human orthologue. Thanks to these features, this nematode has been used for studying the aging process and how it is affected by different substances, as well as to identify the pharmacological targets that can promote healthy aging in humans. Several studies to check the biological effects of phenolic compounds or extracts that are rich in polyphenols have been carried out in *C. elegans*. Among others, these compounds have been found to prolong the lifespan, to promote stress resistance, and to enhance the host innate immunity in the nematode [8,9,10]. Nevertheless, as far as we know, only one study was done with a GP extract that was obtained from Zalema, a Spanish white grape variety. In that study, an increase in the rate of worm survival and significant decreases in the levels of reactive oxygen species (ROS) were observed in *C. elegans* that were submitted to oxidative stress and treated with the pomace extract (100 μg/mL) when compared to the non-treated control worms [11].

In order to improve our knowledge regarding the bioactivity and further valorization of GP, the objective of the present work is to evaluate the biological activity of four polyphenol-rich extracts that were obtained from red GP, using *C. elegans* as an in vivo model and other in vitro antioxidant assays. The chemical composition and in vitro colonic fermentation of the GP extracts used were previously studied [12].

## 2. Material and Methods

### 2.1. Standards and Reagents

2′,7′-Dichlorofluorescein diacetate (DCFH-DA), 2,2′-azobis(2-amidinopropane) dihydrochloride (AAPH), 2,4,6-tris(2-pyridyl)-s-triazine (TPTZ), ampicillin sodium salt, nistatine, agar, yeast extract, fluorodeoxyuridine (FUdR), phosphate-buffered saline (PBS), and cholesterol were purchased from Sigma-Aldrich (Madrid, Spain). 2,2-Azinobis-(3-ethylbenzothiazoline-6-sulfonic acid) diammonium salt (ABTS) and Trolox (6-hydroxy-2,5,7,8-tetramethylchroman-2-carboxylic acid) were purchased from Fluka (Madrid, Spain). Folin reagent, dimethyl sulfoxide (DMSO) and iron trichloride (FeCl_3_·6H_2_O) were obtained from Panreac (Barcelona, Spain), and gallic acid was from Merck (Darmstadt, Germany).

### 2.2. Samples 

The four GP extracts (L1, L2, L3, and L4) that were used in this study, whereby all of them were recorded as Eminol^®^, were industrially obtained by Grupo Matarromera (Valbuena de Duero, Valladolid, Spain) according to the procedure that was explained in Patent ES2319032A1 with some improvements, as described by Gil-Sánchez et al. [12]. Briefly, the raw material for all extracts was the GP that was obtained after the winemaking of red grapes from the Tempranillo variety of *Vitis vinifera* L., harvested from vineyards located in the Ribera de Duero Designation of Origin (Castilla y León, Spain). The extract L4 was obtained from the GP using water as the extraction solvent, whereas for the remaining ones, hydroalcoholic mixtures were used. In the case of L2, the starting GP material had been fermented, and for L1 and L2, a distillation step was also included in the preparation procedure. Maltodextrin was used as encapsulation agents in the spray-drying process for all of the extracts, although for L1 and L2, it was used in combination with silicon dioxide. 

### 2.3. Analysis of Total Phenolic Compounds 

Total phenolic compounds were determined in aqueous solutions (25 mg/mL) of the grape pomace extracts. The samples were shaken vigorously, kept for 30 min in an ultrasonic bath, and then further centrifuged at 2260× *g* for 5 min. The total phenolic content of the solutions was determined using the Folin-Ciocalteu’s reagent, as described by Singleton & Rossi [13]. Absorbance was measured at 760 nm. The results were expressed as mg of gallic acid equivalents (GAE) per g of GP extract by comparison with a standard curve that was prepared with gallic acid (0–4500 µg/mL).

### 2.4. In Vitro Antioxidant Assays

For antioxidant assessment, the four GP extracts L1 to L4 (5 mg) were extracted with 75% methanol that was kept in an ultrasonic bath for 30 min and further centrifuged at 6700× *g* for 5 min; the supernatant was collected and the residue was subjected to the same process three more times. The four supernatants were combined and concentrated under reduced pressure to dryness. The residue was recovered in 1 mL of 75% of methanol. 

FRAP assay. Ferric reducing ability was evaluated according to Benzie and Strain [14], with some modifications. The FRAP reagent contained 10 mM TPTZ solution in 40 mM HCl, 20 mM FeCl_3_·6H_2_O, and acetate buffer (300 mM, pH 3.6) (1:1:10, *v/v/v*). 100 μL of extract was added to 3 mL of the FRAP reagent, and the absorbance was measured at 593 nm after incubation at room temperature for 6 min, using the FRAP reagent as a blank. Different dilutions of the extracts were assayed (equivalent to 0.31–5 mg GP/mL) to check for a linear response, and the results were obtained by interpolating the absorbance on a calibration curve that was obtained with Trolox (30–1000 μM in 75% methanol). Three independent experiments were performed in triplicate for each of the assayed extracts, and the results were expressed as Trolox-equivalent antioxidant capacity (TEAC), here considered as µmol of Trolox with the same antioxidant capacity as 1 mg of the studied extract. 

ABTS/Persulfate assay. The ABTS^•+^ radical was produced by the oxidation of 7 mM ABTS with potassium persulfate (2.45 mM) in water [15]. The mixture was allowed to stand in the dark at room temperature for 16 h before use, and then the ABTS^•+^ solution was diluted with phosphate-buffered saline (PBS) at pH 7.4 to give an absorbance of 0.7 ± 0.02 at 734 nm. 50 μL of extract was mixed with 2 mL of ABTS^•+^ diluted solution and then vortexed for 10 s, and the absorbance was measured at 734 nm after 4 min of reaction at 30 °C. Different dilutions of the extracts were assayed (equivalent to 0.06–1 mg GP/mL) to check for a linear response, and the results were obtained by interpolating the absorbance on a calibration curve that was obtained with Trolox (30–1000 μM in 75% methanol). Three independent experiments were performed in triplicate for each of the assayed extracts, and the results were expressed as Trolox-equivalent antioxidant capacity (TEAC; µmol of Trolox with the same antioxidant capacity as 1 mg of the studied extract).

### 2.5. Assays with C. elegans

#### 2.5.1. Strains and Maintenance Conditions

*C. elegans* strains wild type N2 were obtained from the Caenorhabditis Genetics Centre (CGC) at the University of Minnesota (Minneapolis, MN, USA). Worms were routinely propagated at 20 °C on nematode growth medium (NGM) plates with *Escherichia coli* OP50 strain as a food source. The synchronization of worm cultures was achieved by treating gravid hermaphrodites with bleach:5N NaOH (50:50). The suspension was vortex shaken for one minute and kept a further minute on rest; this process was repeated five times. Eggs were resistant whereas worms were dissolved in the bleach solution. The suspension was centrifuged (2 min, 9500× *g*). The pellet containing the eggs was washed six times with an equal volume of buffer M9 (3 g KH_2_PO_4_, 6 g Na_2_HPO_4_, 5 g NaCl, 1 mL 1 M MgSO_4_, H_2_O to 1 L). The supernatant was then removed and the eggs resuspended and kept in a small volume of M9. Around 100 to 300 μL of the M9 with eggs (depending on eggs concentration) were transferred and incubated in NGM agar plates. The grape pomace extract that was dissolved in DMSO was added to the nematode growth medium during its preparation to get final concentrations in plates among 100 and 1000 μg of grape pomace/mL. NGM plates containing DMSO at the same final concentration as the one used in the assays with extracts (0.1% DMSO, *v/v*) were also prepared and then used as control assays.

In order to evaluate whether the stage of development of the worm had an influence in the resistance against oxidative stress, two different assays were performed, in the first one the animals were recruited for different assays in the fifth day of adulthood and in the second one on the twelfth (aged adulthood). 

#### 2.5.2. Stress Assays

Oxidative stress in worms was induced by subjecting the animals to a temperature of 35 °C that causes damage by the accumulation of ROS [16]. Worms in the first larvae stage were transferred to NGM agar plates (Ø 100 mm) containing the different grape pomace extract from 100 to 1000 μg/mL concentration and cultivated at 20 °C; simultaneous assays were also performed on control plates without pomace extract but with a 0.1% of DMSO. When the worms reached adulthood, they were transferred to new plates with and without pomace extract, but also containing FUdR at a concentration of 150 μM to prevent reproduction and progeny overgrowth. Every two days, the worms were transferred again to fresh plates also containing FUdR and different treatments until they reached the fifth or twelfth day of adulthood, when they were transferred with a platinum wire to agar plates (Ø 35 mm, 20 worms per plate) and then switched to 35 °C for 8 h in an incubator. After that time, the dead and alive nematodes were counted. Assays were performed with approximately 100 nematodes per treatment. Three independent experiments were performed for each extract and conditions were assayed. 

#### 2.5.3. Accumulation of Reactive Oxygen Species (ROS) 

The accumulation of ROS was evaluated at the 5th and 12th day of adult cultivated in the presence and absence of pomace extracts. Cellular ROS were quantified by the dichlorofluorescein (DCF) assay using a microplate reader [17]. Briefly, the worms were individually transferred to the well of a 96-well plate containing 75 μL of PBS and then exposed to thermal stress (2 h at 35 °C), after which 25 μL of DCFH-DA 150 µM solution in ethanol was added to each well. The acetate groups of DCFH-DA were removed in the worm cells, and the released DCFH is oxidized by intracellular ROS to yield the fluorescent dye DCF. The fluorescence from each well was measured immediately after the incorporation of the reagent and every 10 min for 30, using 485 and 535 nm as the excitation and emission wavelengths, respectively. Recording of the DCF fluorescence intensity with time in single worms was used as an index of the individual intracellular levels of ROS. Three independent experiments were performed per treatment, and for each experiment ROS measurements were made in at least 24 individual worms. The measurements were performed in a microplate reader (FLUOstar Omega, BMG labtech, Ortenberg, Germany).

#### 2.5.4. Lifespan Assay

Age synchronized young larvae were transferred to NGM agar plates (Ø 96 mm) containing L3 extract 100 µg/mL and L2 extract 250 µg/mL and control plates with the same percentage of DMSO (0.1%) that the plates with grape pomace extracts. Worms were grown at 20 °C. Once they reached adulthood, they were transferred with a platinum wire to new treatment plates (Ø 35 mm) also containing the assayed compounds and FUdR at a concentration of 150 μM to prevent reproduction and to avoid overlapping generations. This moment was considered to be the first time point for the counting of surviving worms. Twenty animals were transferred per plate. Assays for each compound were performed in at least 100 nematodes. Every two days, worms were transferred to fresh plates also containing FUdR and the different treatments. Worms were scored as dead if they did not respond to touch stimulus with the platinum wire. Three independent assays were carried out for each of the assayed grape pomace extracts.

### 2.6. Statistical Analysis

The statistical analyses were performed using the PC software package SPSS (version 23.0; SPSS Inc., Chicago, IL, USA). ANOVA was applied to make the multiple comparisons of values to determine the possible significant differences between treated and control groups in antioxidant capacity and ROS assays. For lifespan, animal survival was plotted using Kaplan–Meier survival curves and was analyzed by the log-rank test. For analyzing survival to thermal stress, contingency tables were performed and statistical significance was calculated using the Chi Square Test. In every analysis, significant differences were statistically considered at the level of *p* < 0.05.

## 3. Results and Discussion

### 3.1. Chemical and Antioxidant Characterization of the GP Extracts

**Figure 1:**
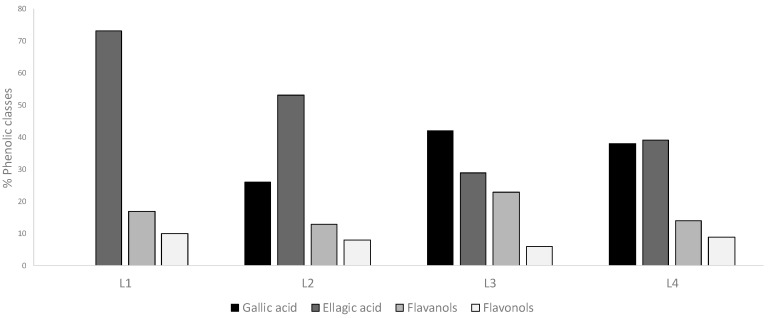
Percentages of distribution of phenolic classes in the studied grape pomace extracts, as calculated from the results that were reported by Gil-Sanchez et al. (2017) [12].

The total extractable polyphenols that were determined in the GP extracts ranged from 26.8 GAE/g (L2 and L4) to 71.8 GAE/g (L3) (Table 1). The individual phenolic composition has been previously analyzed by HPLC, with all of the extracts showing similar phenolic profiles that were characterized by the main presence of gallic acid (GA) and ellagic acid (EA), flavan-3-ols (catechins and procyanidin dimers), and flavonols (quercetin, kaempferol, and myricetin) [12], with the phenolic acids as the majority compounds, with above 70% of the determined components (Figure 1). Regarding other phenolic classes, very minor amounts of anthocyanin pigments were present in extract L3, whereas no stilbenes were detected in any of the studied samples. This is not surprising, since the extracts were obtained from grape pomaces that were obtained after winemaking. As for stilbenes, it is well known that their concentrations in the grape is very low, so that they were not expected to be present in significant amounts in the pomaces either.

The in vitro antioxidant capacity of the four grape extracts was assessed using FRAP and ABTS assays (Table 2). The use of different in vitro methods has been recommended, due to the differences between the various free radical-scavenging assay systems [18]. The ABTS assay measures the ability of a product to scavenge the ABTS^•+^ radical cation. The FRAP assay evaluates the ability to reduce Fe^3+^ to Fe^2+^, therefore determining the reducing capacity of the product [14].

FRAP values ranged from 110 to 530 µmol TE/g among the four pomace extracts, with the highest reducing capacity being exhibited by extract L3, followed by L1, L2, and L4. The ABTS^•+^ scavenging capacity ranged from 300 to 840 μmol TE/g, with the same order among extracts (L3 > L1 > L2 > L4). In all extracts, higher TEAC values were found in the ABTS assay, which was carried out at pH 7.4 against pH 3.6 in the FRAP assay. This is in agreement with the observations of Muzolf et al. [19], which found that the radical scavenging capacity of polyphenols increases with the pH of the medium, due to an increase in the electron donating ability upon deprotonation.

The antioxidant capacity of the pomace extracts should be explained by their phenolic composition. Actually, a good correlation between total phenolic content, as determined by the Folin–Ciocalteu’s reagent and antioxidant capacity values, was observed for the studied extracts (*r* > 0.98). This could be considered to be logical, since the Folin–Ciocalteu’s method is rather a measurement of the reducing ability of a sample than a specific determination of polyphenols. Nevertheless, differences among the results that were obtained for the four extracts in the distinct assays might be attributed not only to their total polyphenol content, but also to their qualitative phenolic composition, as free radical scavenging and metal chelating capacities would be influenced by the number of -OH groups and their position on the phenol rings [20].

Other authors have also reported relevant antioxidant activity for extracts that were obtained from grape and winemaking byproducts [2,21]. However, the comparison among reported data is difficult due to the different analytical methods, standards, units (wet or dry matter basis), grape variety, and extraction methodology. In spite of this difficulty, the antioxidant capacity results that were obtained herein are of the same order of magnitude or slightly higher than those that were previously reported in literature. For example, in the study on the red grape pomaces from Cabernet Sauvignon, Merlot, Bordeaux, and Isabel varieties made by Rockenbach et al. [21], mean values ranging 193–485 µmol TEAC/g were obtained using the ABTS method and 117–249 µmol TEAC/g through the FRAP method.

These good in vitro results, as commented in the hypothesis, must be confirmed using an in vivo model.

### 3.2. Effects of GP Extracts in the Resistance of C. elegans against Oxidative Damage 

To check whether the pomace extracts have any effects on the resistance of *C. elegans* against oxidative damage, the worms were subjected to thermally induced oxidative stress. The stress (35 °C, 8 h) was applied at the fifth and twelfth day of adulthood after being grown in the presence of different concentrations of the pomace extracts ranging from 100 to 1000 μg/mL, and the results were compared with those that were obtained in control worms grown in the absence of extract. 

**Figure 2:**
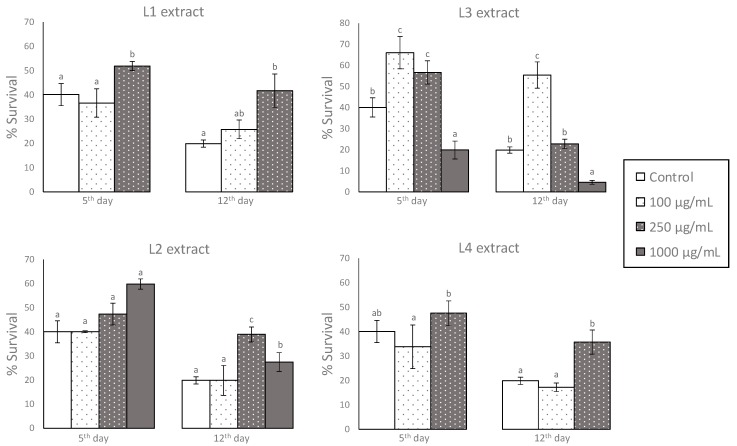
Thermal stress resistances in worms grown in the absence (control) and presence of different concentrations of the pomace extracts. The results are expressed as the percentage of surviving worms after being subjected to thermal stress (35 °C, 8 h) at the 5th day (left side) and 12th day (right side) of adulthood. Three independent experiments were carried out with a minimum of 100 animals per assay. The results are presented as the mean values ± SEM (*n* = 3). Statistical significance was calculated using the Chi Square Test. Different letters in bars indicate significant differences (*p* < 0.05).

At it is shown in Figure 2, the survival rate significantly increased in thermally stressed worms treated with the different GP extracts at some of the studied concentrations in comparison with control worms, whereas, at others, the extracts showed no significant effect; in one case, a decrease in the survival rate relative to untreated worms was even observed (L3 extract, 1000 μg/mL). The optimal extract concentration at which the survival increased was different, depending on the extract. In L3, the survival rate significantly (*p* < 0.05) increased in thermally stressed worms that were treated with 100 μg/mL of pomace extract and 250 μg/mL, but it decreased at 1000 μg/mL in relation to untreated worms. However, L1, L2, and L4 did not increase survival at the lower extract concentrations (100 μg/mL), but they did at 250 μg/mL, although in the case of L2, the increase in the survival rate was only significant in worms that were submitted to stress at day 12th of adulthood. These results might be explained by the different concentration of phenolic compounds existing in the distinct extracts, so that the same concentration of each extract supposed a different amount of phenolic compounds in the culture plates (Table 3). Thus, a protection against oxidative stress would be produced up to a certain level of polyphenols in the culture media, as determined by the composition of the extracts, while high levels would have detrimental effects. In the case of the studied extracts, maximum protection was observed for levels of polyphenols that were around 7 to 9 µg GAE per plate. 

Similar results were also found in a previous study with Zalema grape pomace extracts, where it was also observed that *C. elegans* resistance against thermal stress improved when the worms were grown in the presence of the lower assayed extract concentrations (100 µg/mL), whereas higher concentrations (300 µg/mL) led to a decrease in worm survival [11]. Other authors using polyphenol-rich extracts from different sources, such as tea [22] or cocoa [23], as well as individual pure polyphenols obtained similar observations [8,24,25]. These results pointed to a hormetic response to polyphenols concentration, i.e., an opposed effect induced by low and high doses of a chemical, biological, or physical agent on an organism, as also observed by Saul et al. [26], for some polyphenols, such as ellagic acid or tannic acid. Thus, polyphenols might behave as mild stressors, so that by imposing some degree of oxidative stress, the levels of antioxidant defence might be raised, leading to overall cytoprotection [27,28].

Nonetheless, when analyzing the observed effects, it is necessary to take into account not only the total phenolic concentration, but also the compounds that are present in each case. Gallic acid and ellagic acid are majority compounds in the extracts [12], although with different distributions in each case (Figure 1). Saul et al. evaluated both of the acids regarding their influence on thermal and oxidative stress resistance in *C. elegans* by [26]. According to those authors, ellagic acid was not able to exert stress protection at any concentration level, but it induced adverse effects at elevated levels. However, gallic acid was able to enhance thermal stress resistance at low concentrations, although not significantly, while at higher concentrations, it improved thermal tolerance. Thus, the different amounts of those acids existing in the culture plates, as determined by the phenolic concentration and compound distribution in each extract, might somehow explain the different behavior that was observed in the distinct assays herein.

Flavan-3-ols have been widely related with healthy effects [29,30]. In the studied samples, this group represented 13–22% of the determined polyphenols depending on the extract (Figure 1); whereas, catechin and epicatechin were present in all of the extracts, procyanidin dimers were only found in L3 [12]. Different flavan-3-ols have been evaluated as regards to stress resistance in *C. elegans,* either as pure compounds or as main components in extracts that were obtained from cocoa or green tea. Monomeric catechins were found to enhance the resistance of the worm against thermal and oxidative stress [8,31]. N2 worms that were pretreated with a green tea extract exhibited an increased survival rate after chemically induced oxidative stress with juglone [32], and cocoa extracts were able to protect *C. elegans* from oxidative stress [23]. Flavonols represented a minority group of flavonoids in the studied samples, with around 8% of total polyphenols. Treatment with quercetin increased the survival rates of *C. elegans* under thermal and oxidative stress [24,33]. All in all, it can be supposed that the amounts and distribution of the different phenolics that are present in the GP extracts, as well as the possible synergies among them, should be contributing to the observed effects in the present study. 

An interesting observation in the performed assays was that the protective effects of the GP extracts seemed to be more evident in worms of advanced age (12th day of adulthood) than in younger adult worms (5th day). Thus, for the L2 extract, the increase in the survival rate that was induced at the concentration of 250 µg/mL raised from 7% with respect to untreated controls, when the stress was applied at the 5th day, to 19% when applied at the 12th day. In the L3 extract at a concentration of 100 µg/mL, the increase in the survival rate was 26% on the 5th day and 36% on the 12th day 36%. Similar observations were made in previous assays with catechins, which also increased the resistance against thermal and juglone-induced oxidative stress to a greater extent in older worms [8].

### 3.3. Effects of GP Extracts in the Accumulation of Reactive Oxygen Species (ROS) in C. elegans

**Figure 3:**
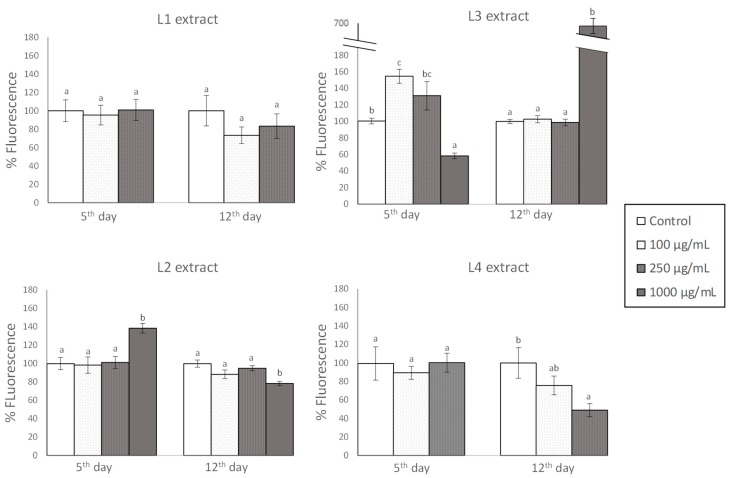
Accumulation of reactive oxygen species (ROS) determined at the 5th day (left side) and the 12th day (right side) of adulthood in worms grown in the presence of different concentrations of the pomace extracts and exposed to thermal stress (2 h at 35 °C). Results are expressed as percentage of fluorescence in relation to control worms that were not treated with grape pomace extracts. The results are presented as the mean values ± SEM (*n* = 3). Statistical significance was calculated using by one-way analysis of variance ANOVA. Different letters in bars indicate significant differences (*p* < 0.05).

Figure 3 shows the levels of ROS in the worms that were submitted to thermal stress after treatment with the different extracts. The results are expressed as percentage of fluorescence relative to controls (worms not treated with pomace extract).

The obtained results are not easy to interpret, as no general pattern of behavior could be concluded regarding the applied treatments (type of extract and doses) with ROS levels. For L1 extract, no significant differences were found between the control and the treated worms, independent of the assay. Similar results were observed for the L4 extract, although the concentration of 250 µg/mL produced a descent in the ROS levels on the 12th day. In general, a slight decrease of ROS levels seemed to exist in the assays at which greater stress resistance was observed (Figure 2). However, such an association was not noticed in L2 and L3 extracts. In the case of L2, an increase in ROS was observed at the highest assayed concentration (1000 µg/mL) on the 5th day, while a decrease was produced at day 12, despite that greater resistance against thermal stress was found in both cases (Figure 2). As for L3, the greater rates of worm survival observed at 100 and 250 µg/mL concentrations (Figure 2) was accompanied by an increase in ROS levels at day 5, but not at day 12 (Figure 3). 

The beneficial health effects of polyphenols have been classically associated to their antioxidant activity. However, as result of their metabolism, in cellular media, they might also give rise to reactive intermediates that can act as prooxidants [28]. Actually, in studies in in vivo models, it has been observed that treatment with different polyphenols can induce an increase in ROS cellular levels [11,34,35]. Whereas, a high concentration of ROS would result in cell damage, exposure to sub-lethal ROS levels might trigger a series of cellular events that may ultimately result in global cytoprotection [3,36,37]. Fu et al. [38] proposed the oxidative fluctuation hypothesis of aging and suggested that the ability of animals to homeostatically maintain the ROS levels within a narrow range is more important for lifespan extension than just minimizing the ROS levels. Other authors also have proposed a model in which the effect on lifespan of a compound that alters ROS levels is a combination of the beneficial and the deleterious effects of ROS in all of the cell types and subcellular sites that are affected by the compound and suggested the existence of an inverted U-shaped relationship between ROS levels and lifespan [39]. Our results suggested that both low and, up to certain point, increased ROS levels could have beneficial effects, which might justify apparently contradictory observations. Obviously, this would not apply for extremely high ROS concentrations, as was produced in the treatment with 1000 µg/mL at day 12 (Figure 3), causing a high degree of oxidative stress that should explain the low rate of worm survival in that assay (Figure 2). This proposal is in accordance with a model in which the *C. elegans* lifespan resulted from a balance between pro-survival ROS-mediated signaling and ROS toxicity [40]. This approach could be explained, since, although ROS are potentially toxic, they are also signaling molecules that modulate different pathways. In fact, the generation of ROS can activate appropriate protective mechanisms through global changes, including in transcriptional patterns that lead to cellular homeostasis. Among others, ROS would act on different signaling pathways that are involved in responding to cellular stress, damage repair, and even the intrinsic apoptotic pathway, which contributes to organismal homeostasis by eliminating defective or unwanted cells [39,41]. For instance, it is known that light prooxidant changes in intracellular redox tone that are produced by phytochemicals might trigger the Keap-1/Nrf2–ARE signaling pathway and the consequent activation of different protective mechanisms [42]. Besides, numerous studies have demonstrated the influence of different polyphenols in other different signaling pathways, such as insulin/IGF-1 signaling pathway [34], JNK (c-Jun amino-terminal kinase) [43], and p38-MAP kinase [10]. Therefore, the study of the components of different pathways may provide a powerful entry for understanding the molecular mechanism of action of polyphenols.

### 3.4. Effects of GP Extracts in C. elegans Lifespan

**Figure 4:**
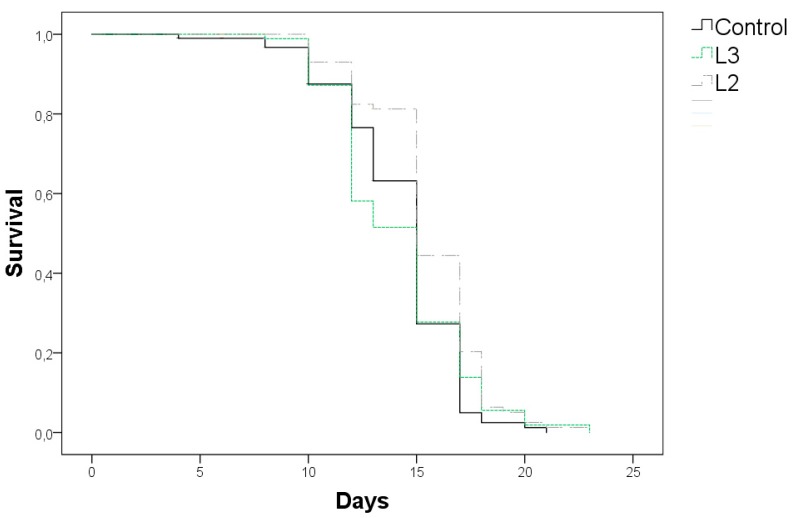
Survival curves of *C. elegans* wild-type N2 strain grown in culture media supplemented with L3 (100 µg/mL) or L2 extracts (250 µg/mL), compared to a control of untreated worms.

The influence on worm lifespan was studied only for L2 and L3, which presented the lowest and highest polyphenol concentrations among the studied extracts. Assays were performed at concentrations of 100 µg/mL for L3 (7.13 µg GAE/plate) and 250 µg/mL for L2 (6.70 µg GAE/plate) in the culture media, as they were the concentrations for which more accused influence on stress resistance was observed for those extracts. Figure 4 shows the curves of survival that were obtained at 20 °C for L2 and L3 as compared with the control untreated worms. The calculated mean and maximum lifespan (determined as the longest living 10% of each population) of the worms in those assays are presented in Table 4. 

A significant increase was noted in mean and maximum life duration (15.6 days and 19.5 days, respectively) in worms that were grown in the presence of L2 extract at the assayed concentration with regard to the untreated controls (14.3 days and 17.7 days). However, an increase in the maximum life span (19.1 days), but no significant influence on mean life duration (14.2 days), was observed for L3, despite that this extract showed as the most active one in the previous thermal stress assay. These results seemed to suggest that L2 showed more positive effects on worms in normal life conditions, whereas the effect of L3 would be more accused in stress conditions, which might be related to the phenolic composition of the samples. 

No information regarding the effects on *C. elegans* lifespan of GP extracts has been found in the literature. However, different individual phenolics (tannic acid, gallic acid, ellagic acid, and catechin), some of which were majority compounds in our extracts, were checked by Saul et al. [26] regarding their effects on lifespan, stress resistance, and antioxidant capacity in *C. elegans*. Those authors found that, despite that all of them were able to extend worm lifespan, the individual impact and background mechanisms were different in each case. Tannic acid and ellagic acid efficiently increased lifespan at low doses but they acted in a hormetic manner displaying detrimental effects at higher concentrations in the assayed range (50 to 800 µM), whereas gallic acid and catechin were able to prolong mean lifespan in relation to controls over the range of assayed concentrations. Furthermore, gallic acid, tannic acid, and, in lesser extent, catechin, enhanced resistance against thermal stress (35 °C, 8 h), but only catechin showed significant antioxidant protection. On the other hand, no apparent correlation was observed between the antioxidant capacity of the assayed compounds and lifespan extension [26]. In our study, both L2 and L3 showed phenolic acids as majority compounds, representing 78% and 71% of the determined phenolic compounds, followed by flavan-3-ols (13% and 23%) and flavonols (8% and 6%) (Figure 1); however, whereas ellagic acid was the majority in L2 (53% of total analyzed polyphenols), gallic acid was the main one in L3 (42% of total analyzed polyphenols). Thus, based on Saul et al. [26] observations, the positive effects on worm lifespan that were observed for L2 at the assayed concentration might be related to its richness in phenolic acids, and especially ellagic acid, while the greater resistance against thermal stress induced by L3 might be due to its higher contents of gallic acid and flavan-3-ols (catechins and procyanidins). Regarding catechins, different observations have been made by different authors concerning their influence on *C. elegans* lifespan. While some authors found an increase in mean life duration in worms that were treated with catechin or epicatechin (100 to 800 µM) [26,31,44], others observed no effects in lifespan [8,45] and found that, despite no extension in mean being produced in worms that were treated with epicatechin (200 µM), this compound was able to increase the maximum lifespan determined as the longest living 10% population, which is in agreement with the results that were obtained herein for L3 extract. As for flavonols, the minority group of phenolics in our samples, an enhancement in life duration has been observed by different authors in studies that were carried out with different compounds, such as quercetin, isorhamnetin, myricetin, or kaempferol [24,33,46], as characterized by a hormetic response in the case of quercetin [25], which might also support, at least in part, the observations made herein.

## 4. Conclusions

The studied GP extracts displayed capacity to increase the resistance against thermally induced oxidative stress when used at relatively low concentrations, but they may be detrimental at higher levels, indicating a hormetic response. However, the ability to improve stress resistance was not strictly related to the life extending properties of the extracts. Actually, no extension in mean lifespan was observed for the most active extract (L3) regarding protection against thermal stress, although it was able to extend maximum life duration, which might suggest greater effects in more aged individuals. Moreover, the influence of ROS level was, however, uncertain, although the results pointed that moderate changes in the ROS level could have beneficial effects and might lead to a compensatory response. However, an extreme increase or descent of ROS levels produce detrimental results, and in this case reflected in a descent of survival under stress conditions.

## Figures and Tables

**Table 1:** Concentration of total phenolic compounds in the different grape pomace extracts.

Total Polyphenols (mg Gallic Acid Equivalents/g dw)	
**L1**	37.4
**L2**	26.8
**L3**	71.3
**L4**	26.8

**Table 2:** Antioxidant activity in grape pomace (GP) extracts. Data are expressed as mean ± standard deviation (*n* = 3). Means followed by the same letter within a column indicate no significant (*p* > 0.05) difference among samples. Trolox-equivalent antioxidant capacity (TEAC) values: µmol TE/g dry extract).

	ABTS (µmol TE/g Dry Extract)	FRAP (µmol TE/g Dry Extract)
**L1**	420 ± 10 ^b^	220 ± 20 ^b^
**L2**	400 ± 20 ^b^	170 ± 20 ^b^
**L3**	840 ± 20 ^a^	530 ± 60 ^a^
**L4**	300 ± 50 ^b^	110 ± 10 ^b^

**Table 3:** Amount of phenolic compound per plate for different concentrations of each tested samples estimated from their total polyphenol values determined by the Folin–Ciocalteu.

	Polyphenols per Plate			
Concentration of Extracts per Plate	L1	L2	L3	L4
100 µg/mL	3.74 µg GAE	2.68 µg GAE	7.13 µg GAE	2.68 µg GAE
250 µg/mL	9.36 µg GAE	6.70 µg GAE	17.83 µg GAE	6.70 µg GAE
1000 µg/mL	-	26.79 µg GAE	71.30 µg GAE	-

**Table 4:** Influence of L3 extract 100 µg/mL and L2 extract 250 µg/mL in the culture medium on the lifespan of wild-type N2 *C. elegans* under normal grown conditions at 20 °C.

Treatment	Mean (Days) ^a^	*p* vs. Control (Log-Rank)	Maximun 10% (Days) ^b^	*p* vs. Control (Anova)
Control	14.3 ± 0.30		17.7 ± 1.26	
L3 100 µg/mL	14.2 ± 0.35	0.96	19.1 ± 1.52	0.047
L2 250 µg/mL	15.6 ± 0.18	0.00	19.5 ± 1.41	0.007

^a^ Mean ± standard deviation. Statistical significance was calculated by log-rank testing. ^b^ Maximum lifespan ± standard deviation was determined as the mean life span of longest living 10% of each population. Statistical significance was calculated using by one-way analysis of variance ANOVA. The differences of both analyses were considered significant at (*p* < 0.05).
